# Exposure to Melan-A/MART-1_26-35_ tumor epitope specific CD8^+^T cells reveals immune escape by affecting the ubiquitin-proteasome system (UPS)

**DOI:** 10.1038/srep25208

**Published:** 2016-05-04

**Authors:** Frédéric Ebstein, Martin Keller, Annette Paschen, Peter Walden, Michael Seeger, Elke Bürger, Elke Krüger, Dirk Schadendorf, Peter-M. Kloetzel, Ulrike Seifert

**Affiliations:** 1 Charité-Universitätsmedizin Berlin, Institut für Biochemie, Charité-Platz 1/ Virchowweg 6, 10117 Berlin, Germany; 2 Department of Dermatology, University Hospital, University Duisburg-Essen and German Cancer Consortium (DKTK), Hufelandstr. 55, 45122 Essen, Germany; 3 Charité-Universitätsmedizin Berlin, Klinik für Dermatologie, Venerologie und Allergologie, Charité Platz 1, 10117 Berlin, Germany; 4 Institut für Molekulare und Klinische Immunologie, Medizinische Fakultät der Otto-von-Guericke-Universität Magdeburg, Leipzigerstr. 44, 39120 Magdeburg, Germany; 5 Berlin Institute of Health Kapelle-Ufer 2 10117 Berlin, Germany

## Abstract

Efficient processing of target antigens by the ubiquitin-proteasome-system (UPS) is essential for treatment of cancers by T cell therapies. However, immune escape due to altered expression of IFN-γ-inducible components of the antigen presentation machinery and consequent inefficient processing of HLA-dependent tumor epitopes can be one important reason for failure of such therapies. Here, we show that short-term co-culture of Melan-A/MART-1 tumor antigen-expressing melanoma cells with Melan-A/MART-1_26-35_-specific cytotoxic T lymphocytes (CTL) led to resistance against CTL-induced lysis because of impaired Melan-A/MART-1_26-35_ epitope processing. Interestingly, deregulation of p97/VCP expression, which is an IFN-γ-independent component of the UPS and part of the ER-dependent protein degradation pathway (ERAD), was found to be essentially involved in the observed immune escape. In support, our data demonstrate that re-expression of p97/VCP in Melan-A/MART-1_26-35_ CTL-resistant melanoma cells completely restored immune recognition by Melan-A/MART-1_26-35_ CTL. In conclusion, our experiments show that impaired expression of IFN-γ-independent components of the UPS can exert rapid immune evasion of tumor cells and suggest that tumor antigens processed by distinct UPS degradation pathways should be simultaneously targeted in T cell therapies to restrict the likelihood of immune evasion due to impaired antigen processing.

The generation of antitumor cytotoxic T cell (CTL) response involves the processing and presentation of tumor antigens onto MHC class I molecules^1^,^2^. These specialized T cells can detect target cells that endogenously express protein molecules (i.e. mutated, over-expressed and/or tissue differentiation antigens) and subsequently remove these cells from the body^3^,^4^. The vast majority of peptides presented by MHC class I molecules at the cell surface for recognition by specific cytotoxic T-cells (CTL) is generated by the ubiquitin-proteasome system (UPS) with its central multicatalytic proteinase complex, the proteasome^5^,^6^. Peptides generated by the proteasome system are transported by TAP proteins (transporter associated with antigen presentation) into the ER where peptides of appropriate length and affinity will bind to MHC class I proteins to be presented at the cell surface for immune recognition by CTL^7^,^8^,^9^.

The standard 20S proteasome (s-20S proteasome) with its active site β-subunits β1, β2 and β5 represents the central catalytic unit of the UPS and the catalytic core of the 30S proteasome which is built by the association of two 19S regulator complexes with the 20S core complex. IFN-γ induces the synthesis of alternative catalytic immunosubunits (i-subunits), i.e. β1i/LMP2, β2i/MECL1 and β5i/LMP7 and the concomitant formation of immunoproteasome (i-proteasome) subtypes^8^,^9^,^10^. The 30S proteasome complexes are responsible for the degradation of proteins in the nucleus and the cytosol, which are marked for degradation by a poly-ubiquitin chain and consequently recognized by specific subunits of the 19S regulator complex. A special problem arises for the degradation and processing of membrane proteins, which are co-translationally transported into the endoplasmic reticulum (ER). These proteins, if misfolded or mutated, are re-translocated to the cytosolic side of the ER to be degraded by the 30S proteasome complex in an ubiquitin-dependent manner^11^,^12^,^13^. This process is called ER associated degradation pathway (ERAD) and essentially requires the so-called ERAD-complex within the ER-membrane. This complex is composed of a number of different proteins including Derlin, VIMP, Herp and the E3-ligase HRD1^14^,^15^. Functionally associated with the ERAD pathway on the cytosolic site of the ER is the p97/VCP ATPase complex. The p97/VCP complex binds and extracts poly-ubiquitinated proteins from the membrane making them available for proteasomal degradation at the cytosolic site of the ER^16^,^17^.

Efficient processing and generation of the target antigenic peptides by the UPS is essential for treatment of cancers by T-cell therapy. However, immune escape due to inefficient processing of HLA dependent tumor epitopes can be one important reason for failure of such therapies. It is known that tumors can down-regulate or completely lose expression of tumor antigens and HLA class I molecules, thereby escaping from T cell recognition^18^,^19^. Modulation of the UPS has also been observed and, in particular, the expression of the IFN-γ inducible components of the UPS such as PA28α/β and the i-subunits β1i/LMP2 and β5i/LMP7 were found to be altered in tumor cells, affecting both the quantity and in certain cases also the quality of the generated epitopes^20^,^21^,^22^. In some cases, a deficient expression of proteasome components could be reverted in the presence of IFN-γ, thereby also reconstituting MHC class I surface expression^23^. However, due to the complexity of the UPS and its associated pathways, only a few immune escape mechanisms have been characterized so far, although knowledge of these mechanisms is a prerequisite for the improvement of cancer immunotherapy.

To identify novel mechanisms by which tumors can become refractory to immune elimination, human melanoma cells expressing the transmembrane Melan-A/MART-1 tumor antigen were exposed to two rounds of brief co-culture with Melan-A/MART-1_26-35_-specific CTL. Immune selected melanoma cell clones, being resistant to lysis by Melan-A/MART-1_26-35_ CTL were investigated for the underlying mechanism focusing on the role of the proteasomal antigen processing machinery. We detected a deregulated ERAD pathway as a so far unknown mechanism for immune escape of melanoma cells. In particular, the essential non-inducible ERAD component p97/VCP has been found to be down regulated in CTL-resistant melanoma cells. Rescue experiments reconstituting p97/VCP expression in melanoma cells resulted in enhanced Melan-A/MART-1_26-35_ epitope recognition, underlining the *in vivo* functional relevance of our results.

## Results

### Selection of melanoma cell clones resistant to CTL-mediated lysis by Melan-A/MART-1_26-35_-specific CTL

**Figure 1:**
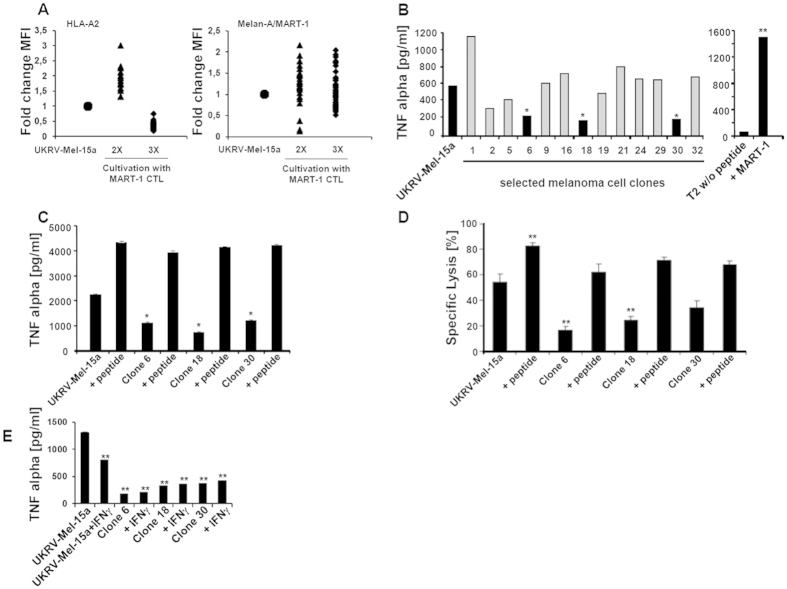
Analysis of melanoma cell clones resistant to MART-1_26-35_-specific CTL lysis. (**A**) Immune selected melanoma cell clones derived from co-cultivation of UKRV-Mel-15a cells (Mel 15) with Melan-A/MART-1_26-35_ specific CTL were analyzed for HLA-A*0201 and MART-1 expression by flow cytometry after two (triangle) and three (rhomb) selection cycles. Data are presented as fold changes compared to the parental UKRV-Mel-15a cell line (circle) whose HLA-A*0201 and Melan-A/MART-1 expression levels were set to 1. (**B**) UKRV-Mel-15a clones obtained after two rounds of immune selection, were tested for their ability to present the Melan-A/MART-1_26-35_ epitope. UKRV-Mel-15a clones were co-cultured for 6 h with Melan-A/MART-1_26-35_ specific CTL, whose activation was determined by measuring the TNF-α-content in the supernatants by ELISA. The UKRV-Mel-15a clones 6, 18 and 30 (black columns) exhibited a strongly reduced capacity of presenting Melan-A/MART-1_26-35_ (*p < 0.05, Student’s *t* test) and were therefore selected for further analysis. T2 cells loaded with 5 μM of the Melan-A/MART-1_26-35_ synthetic peptide were included as positive control (right panel). **p < 0.01 *vs* unloaded T2 cells (Student’s *t* test). (**C**) The comparability of the HLA-A*0201 expression level across the three immune selected UKRV-Mel-15a clones was further verified by analyzing their capacity to stimulate Melan-A/MART-1_26-35_ specific CTL when loaded with 5 μM of the Melan-A/MART-1_26-35_ peptide. CTL activation was determined in a 6-h TNF-α release assay. Error bars represent standard deviation (n = 2). *p < 0.05 *vs* UKRV-Mel-15a parental cells (Student’s *t* test). (**D**) The sensitivity of the three UKRV-Mel-15a clones to lysis by Melan-A/MART-1_26-35_-specific CTL was tested in a^51^ Cr release assay (E:T 10:1). Positive control consisted in UKRV-Mel-15a clones 6, 18 and 30 exogenously pulsed with 5 μM of the Melan-A/MART-1_26-35_ peptide. **p < 0.01 *vs* the UKRV-Mel-15a parental cell line (Student’s *t* test). (**E**) The effect of IFN-γ on the presentation of Melan-A/MART-1_26-35_ by the UKRV-Mel-15a clones 6, 18 and 30 was evaluated in a 6-h TNF-α CTL assay. To this end, cells were treated with IFN-γ at 200 U/ml for 48 h prior to an exposure to Melan-A/MART-1_26-35_ CTL and measurement of the released TNF-α by ELISA. Error bars represent standard deviation (n = 2). **p < 0.01 *vs* UKRV-Mel-15a parental cells (Student’s *t* test). The experiments shown are representative of at least two.

To identify unknown immune evasion mechanisms allowing melanoma cells to circumvent recognition by specific CTL, the human HLA-A^*^0201-positive melanoma cell line UKRV-Mel-15a expressing the transmembrane tumor antigen Melan-A/MART-1^20^,^24^,^25^ was co-incubated with an HLA-A*0201-restricted Melan-A/MART-1_26-35_-specific CTL clone. Surviving UKRV-Mel-15a cells were exposed to two or three rounds of CTL co-culture selecting for resistant tumor cell clones. To exclude resistance due to HLA or antigen down-regulation, UKRV-Mel-15a cells were analyzed for HLA-A*0201 and Melan-A/MART-1 expression by flow cytometry. As shown in Fig. 1A, three rounds of co-culture resulted in considerable HLA-A*0201 down-regulation. We therefore decided to apply only two rounds of CTL co-culture for the selection of resistant melanoma clones, because under these conditions the HLA-A*0201 and Melan-A/MART-1 expressions of the surviving clones was similar or even higher than those of the parental UKRV-Mel-15a cell line (Fig. 1A).

We next tested the ability of the UKRV-Mel-15a derived clones to present the Melan-A/MART-1_26-35_ epitope by exposing them to Melan-A/MART-1_26-35_-specific CTL in a 6-h antigen presentation TNF-α assay. Exposing Melan-A/MART-1_26-35_ CTL to peptide-pulsed T2 cells but not unloaded T2 cells resulted in increased production of TNF-α, thereby confirming the specificity of our read-out system (Fig. 1B, right panel). Of more than 30 UKRV-Mel-15a clones, only the clones 6, 18 and 30 exhibited significantly impaired recognition by Melan-A/MART-1_26-35_-specific CTL and therefore were chosen for further analysis (Fig. 1B). Importantly, both TNF-α release and cytolysis by Melan-A/MART-1_26-35_-specific CTL could be restored upon exogenous loading of UKRV-Mel-15a clones with synthetic Melan-A/MART-1_26-35_ peptide (Fig. 1C,D). This demonstrates that the HLA-A^*^0201 expression level on these clones was not rate limiting for immune recognition and that the observed immune escape was not due to resistance of the three selected melanoma cell clones against CTL-induced cytolysis.

Down-regulation of constituents of the antigen processing machinery in tumor cells and concomitant impaired epitope generation and presentation is a well-known evasion mechanism described for a variety of different human tumors^22^,^23^,^26^,^27^. However, as mentioned above, the expression of these components can be restored following IFN-γ treatment. Importantly, the expression levels of TAP1 and TAP2 as well as of the three proteasome immunosubunits in UKRV-Mel-15a clones were comparable to those of the non-selected parental UKRV-Mel-15a cells (Fig. S1). Only the clone 30 exhibited some impairment in the inducible expression of the i-subunits, as evidenced by its incapacity to up-regulate β1i/LMP2, β2i/MECL and β5i/LMP7 in response to IFN-γ. This suggests that the observed resistance of the selected UKRV-Mel-15a clones to CTL-mediated lysis was not due to an altered IFN-γ signaling pathway. This notion is supported by the observation that IFN-γ exposure failed to modify the phenotype of the UKRV-Mel-15a clones 6, 18 and 30 (Fig. 1E).

### Deregulation of ERAD components in resistant melanoma cell clones

**Figure 2:**
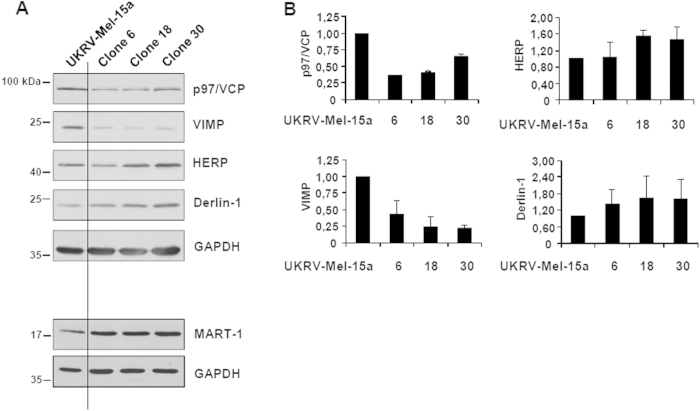
The expression levels of components of the ERAD pathway are altered in immune selected UKRV-Mel-15a cell clones. (**A**) The UKRV-Mel-15a clones 6, 18 and 30 as well as the UKRV-Mel-15a parental cell line were subjected to protein extraction. Whole cell extracts were resolved by SDS-PAGE followed by Western blotting using antibodies specific for p97/VCP, VIMP, HERP, Melan-A/MART-1 and Derlin-1, as indicated. Equal protein loading was ensured by probing the membrane with anti-GAPDH antibody. (**B**) Fold changes of the expression levels of p97/VCP, HERP, VIMP and Derlin-1 between the UKRV-Mel-15a parental cell line and the UKRV-Mel-15a clones 6, 18 and 30, as visualized by densitometric analysis. The band intensity of each protein was normalized to that of GAPDH and subsequently expressed as fold-changes compared to the UKRV-Mel-15a parental cell line which was set at 1.

To determine which of the UPS components and its associated pathways was responsible for the down-regulation of Melan-A/MART-1_26-35_ epitope presentation; UKRV-Mel-15a clones were subjected to microarray gene expression analysis focusing on more than 1000 components of the antigen processing machinery. Interestingly, all three selected UKRV-Mel-15a clones displayed considerable alterations in the expression levels of a set of mRNAs encoding p97/VCP, VIMP, HERP and Derlin-1 (Table 1) and thus proteins whose functions are related to or directly connected with the ER-dependent degradation (ERAD) pathway^28^. Because cellular protein levels strongly depend on the rate of synthesis and their half-lives, expression levels of mRNAs and corresponding detectable protein levels do not necessarily correlate. Therefore, we next analyzed the expression levels of the affected ERAD-associated proteins by Western blot. As shown in Fig. 2A,B, all three resistant UKRV-Mel-15a clones revealed a strong down-regulation of the ERAD components p97/VCP, which is essential for the extraction of poly-ubiquitinated substrates from the ER^29^, and VIMP, which is a seleno-protein associated with p97/VCP as well as with the membrane protein Derlin-1^17^. In contrast, slightly increased expression levels were found for HERP and Derlin-1 (Fig. 2A,B). Importantly, there was no substantial loss of the Melan-A/MART-1 antigen expression in the UKRV-Mel-15a clones 6, 18 and 30. These data therefore suggested that deregulated expression of ERAD components might be causative for the impaired Melan-A/MART-1_26-35_ epitope generation and presentation by the three UKRV-Mel-15a clones.

### p97/VCP is a prerequisite for Melan-A/MART-1_26-35_ epitope generation

**Figure 3:**
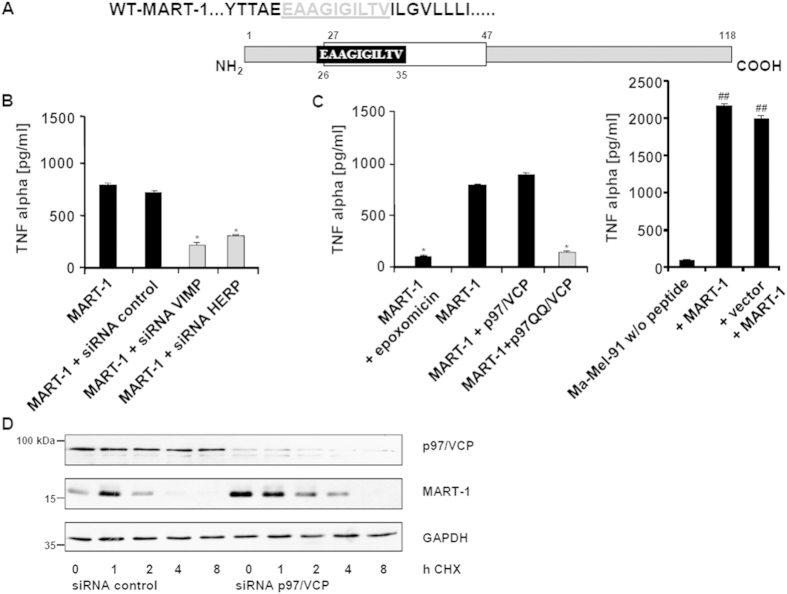
Melan-A/MART-1_26-35_ epitope presentation is ERAD-dependent. (**A**) Schematic representation of the Melan-A/MART-1_26-35_ epitope which is entirely localized within the transmembrane domain, except for the first amino acid residue. (**B**) The melanoma cell line Ma-Mel-91 (Melan-A/MART-1^−^, HLA-A*0201) was exposed to non-targeting (control) siRNA or siRNA directed against VIMP or HERP1 prior to a subsequent transfection with an expression vector encoding the wild-type full-length Melan-A/MART-1 tumor antigen. The ability of these cells to present the Melan-A/MART-1_26-35_ epitope was evaluated in a 6 h TNF-α release assay upon exposure to Melan-A/MART-1_26-35_ CTL. The content of TNF-α in the supernatants was measured by ELISA. *p < 0.05 *vs* Ma-Mel-91 cells expressing Melan-A/MART-1 and exposed to control siRNA (Student’s *t* test) (**C**) The impact of p97/VCP on Melan-A/MART-1_26-35_ presentation was evaluated by transfecting Ma-Mel-91 cells with Melan-A/MART-1 in combination with either wild-type p97/VCP or a dominant negative mutant of p97/VCP (p97QQ). After 24 h of transfection, the cells were tested for their capacity to present the Melan-A/MART-1_26-35_ antigenic peptide by cultivating them in the presence of Melan-A/MART-1_26-35_ CTL and followed by subsequent measurement of TNF-α by ELISA. In addition, proteasome dependency of Melan-A/MART-1_26-35_ epitope generation was demonstrated by treating the Ma-Mel-91 cells transfected with the Melan-A/MART-1 expression plasmid with 1 μM of the proteasome inhibitor epoxomicin for 4 h. As control, Ma-Mel-91 cells were loaded with the Melan-A/MART-1_26-35_ synthetic peptide or were transfected with the pcDNA3.1 plasmid and loaded with the Melan-A/MART-1 peptide. *p < 0.05 *vs* Melan-A/MART-1-expressing Ma-Mel-91 cells and ^##^*p* < 0.01 *vs* unloaded Ma-Mel-91 cells (Student’s *t* test) (**D**) Silencing of p97/VCP by siRNA results in stabilization of the Melan-A/MART-1 protein expression. HeLa cells were exposed to either non-targeting (control) or p97/VCP siRNA for 24 h prior to a subsequent 24 h transfection with an expression plasmid encoding wild-type Melan-A/MART-1 and subjected to CHX-based chase assay. Cells were collected after 1, 2, 4 and 8 h and Western blot analysis was performed for each time point using antibodies specific for Melan-A/MART-1, p97/VCP and GAPDH (loading control).

Apart from the first N-terminal amino acid residue which is directed to the luminal compartment, the entire Melan-A/MART-1_26-35_ 10-mer epitope is embedded within the Melan-A/MART-1 transmembrane domain^30^ (Fig. 3A). Therefore, for proteasome-dependent antigen processing, the Melan-A/MART-1 protein must be retro-translocated into the cytosol. We next aimed to evaluate the impact of VIMP and HERP on Melan-A/MART-1_26-35_ epitope generation independently of the Melan-A/MART-1 endogenous expression level. To this end, both of these ERAD components were knocked down by siRNA in the Ma-Mel-91-melanoma cell line (Melan-A/MART-1^−^/HLA-A^*^0201^+^) which was subsequently engineered to express the Melan-A/MART-1 antigen. Strikingly and as illustrated in Fig. 3B, gene silencing of either VIMP or HERP resulted in a substantial reduced presentation of the Melan-A/MART-1_26-35_ epitope. Because p97/VCP expression was also found to be significantly reduced in our selected UKRV-Mel-15a clones 6, 18 and 30, we next tested its involvement in Melan-A/MART-1_26-35_ epitope generation by expressing a dominant negative mutant of p97/VCP (p97QQ), which abrogates cellular p97/VCP function^16^. Interestingly, although the expression of p97QQ had virtually no effect on cell viability (data not shown), it almost completely abrogated Melan-A/MART-1_26-35_ epitope generation, whose levels, under these conditions, were quite similar to those observed when proteasomes were inhibited with epoxomicin (Fig. 3C). Accordingly, CHX-based chase experiments show that p97/VCP silencing resulted in a significant stabilization of the Melan-A/MART-1 protein (Fig. 3D). It is to note, that neither the down-regulation of VIMP or HERP by siRNA nor the plasmid-mediated transfection of p97/VCP or p97QQ substantially affected the expression level of the Melan-A/MART-1 full-length protein (Fig. S2).

### Melan-A/MART-1_26-35_ epitope recognition is rescued by overexpression p97/VCP

The observation, that the inhibition and/or gene silencing of VIMP and p97/VCP almost completely abrogated Melan-A/MART-1_26-35_ epitope presentation and that both proteins were found to be down-regulated in CTL-resistant UKRV-Mel-15a clones suggests that both or one of them may be causative for the observed immune evasion. Therefore, we next sought to determine whether the immune escape of the UKRV-Mel-15a clones was reversible by restoring p97/VCP or VIMP expression.

**Figure 4:**
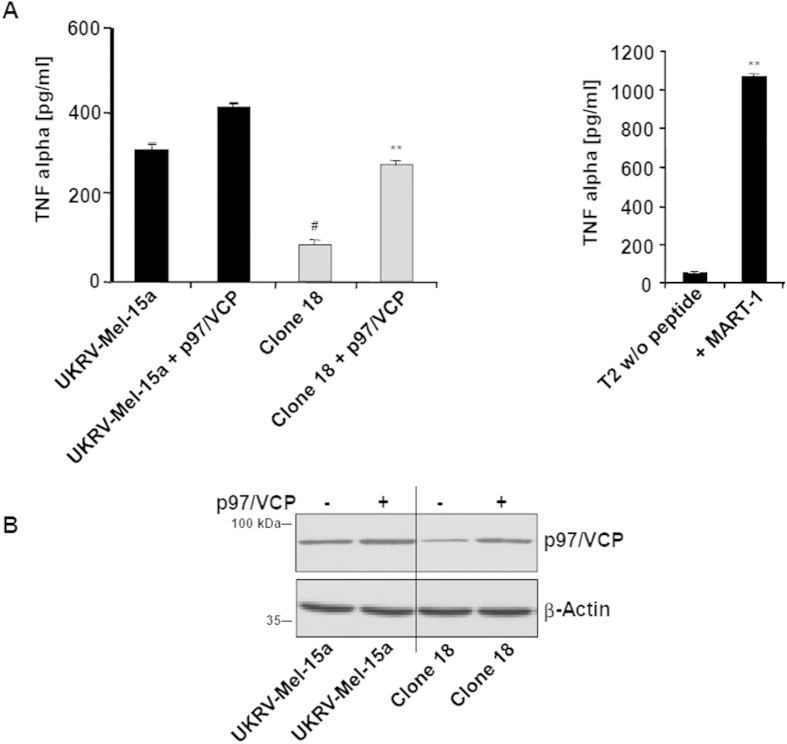
Re-expression of p97/VCP reconstitutes Melan-A/MART-1_26-35_ epitope presentation. (**A**) The UKRV-Mel-15a clone 18 as well as the UKRV-Mel-15a parental cells were transfected with an expression vector encoding the p97/VCP full-length protein, as indicated. After 24 h of transfection, cells were exposed to Melan-A/MART-1_26-35_ CTL to evaluate their capacity to present the Melan-A/MART-1_26-35_ epitope. Following a 6 h co-culture, the supernatants were harvested and tested for their TNF-α content by ELISA. Controls in this experiment consisted of unloaded T2 and T2 cells loaded with 5 μM of the Melan-A/MART-1_26-35_ synthetic peptide (right panel). Shown is one representative experiment out of three. **p < 0.01 *vs* UKRV-Mel-15a clone 18 (left graph) or unloaded T2 cells (right graph) and ^#^*p* < 0.05 *vs* UKRV-Mel-15a cells (Student’s *t* test). (**B**) Melanoma cells were subjected to protein extraction and whole cell extracts were resolved on SDS-PAGE followed by Western blotting using antibodies specific for p97/VCP and β-actin (loading control), as indicated.

**Figure 5:**
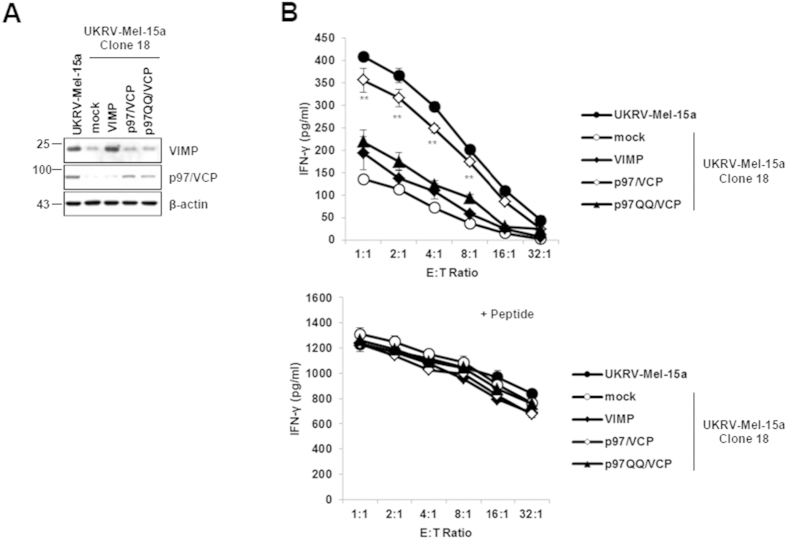
Melan-A/MART-1_26-35_ epitope presentation in the immune challenged UKRV-Mel-15a clone 18 is not rescued by VIMP over-expression. (**A**) The UKRV-Mel-15a clone 18 was subjected to a 24-h transfection with an empty vector (mock) or expression vectors encoding VIMP, p97/VCP or p97QQ/VCP, as indicated. Whole cell-extracts were resolved on 15% SDS-PAGE and analysed by western-blotting using antibodies against VIMP, p97/VCP and β-actin (loading control). (**B**) The ability of the UKRV-Mel-15a clone 18 over-expressing VIMP, p97/VCP or p97QQ/VCP to present the Melan-A/MART-1_26-35_ antigenic peptide was evaluated by exposing them to Melan-A/MART-1_26-35_-specific CTL followed by IFN-γ measurement in the supernatants by ELISA. In addition, cells were pulsed with 1 μM of the Melan-A/MART-1_26-35_ synthetic peptide, as a positive control. Shown is one representative experiment out of two. **p < 0.01 *vs* UKRV-Mel-15a clone 18 (mock) (Student’s *t* test).

To this end, we performed MHC class I presentation rescue experiments by transfecting the UKRV-Mel-15a clone 18 with expression vectors encoding full-length p97/VCP or VIMP proteins. As shown in Fig. 4A,B, re-expression of p97/VCP restored stimulation of Melan-A/MART-1_26-35_-specific T cells to levels comparable to those detected upon exposure to the parental UKVR-Mel-15a cell line, demonstrating that the observed down-regulated expression of p97/VPC was indeed responsible for the observed immune escape of the UKRV-Mel-15a Mel-15 clones. Accordingly, RNAi-mediated down-regulation of p97/VCP in the parental UKVR-Mel-15a cell line was accompanied by severe impairment of Melan-A/MART-1_26-35_ presentation (Fig. S3), further confirming that p97/VCP ensures the supply of Melan-A/MART-1_26-35_ antigenic peptides under normal conditions. This also suggests that the observed emergence of resistant clones following Melan-A/MART-1_26-35_ CTL exposure is most likely driven by the spontaneous loss of p97/VCP expression in the heterogeneous UKRV-Mel-15a parental melanoma cell population or a selective enrichment of cells expressing low levels of p97/VCP. In contrast, over-expression of the p97/VCP recruiting membrane protein VIMP failed to rescue the Melan-A/MART-1_26-35_ epitope presentation (Fig. 5A,B). This, however, is in agreement with a previous report showing that the over-expression of VIMP in COS cells alters ER morphology which may cause dysfunction of the ERAD pathway^17^.

### Down-regulation of p97/VCP is also an immune escape mechanism in Ma-Mel-63a cells

**Figure 6:**
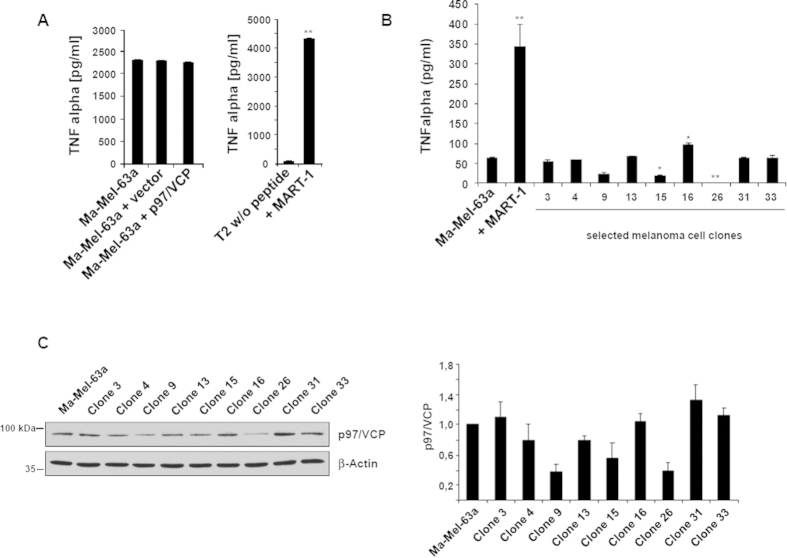
Analysis of Ma-Mel-63a melanoma cell clones resistant to Melan-A/MART-1_26-35_-specific CTL lysis. (**A**) The Ma-Mel-63a cells (Melan-A/MART-1^+^, HLA-A*0201^+^) were tested for their ability to endogenously process and present the Melan-A/MART-1_26-35_ antigenic peptide by cultivating them with Melan-A/MART-1_26-35_ CTL. Additionally, Ma-Mel-63a cells were either transfected with pcDNA3.1 plasmid or an expression vector encoding the p97/VCP full-length protein. After 6 h, supernatants were harvested and their content for TNF-α was determined by ELISA. Controls in this assay consisted in non-pulsed T2 cells (negative) and T2 cells pulsed with 5 μM of the 10-mer synthetic peptide (right panel). **p < 0.01 *vs* unloaded T2 cells (Student’s *t* test) (**B**) The Ma-Mel-63a clones 3,4,9,13,15,16,26,31 and 33 were tested for their ability to present the Melan-A/MART-1_26-35_ epitope by culturing them with Melan-A/MART-1_26-35_-specific CTL. Activation of CTL was assessed by measuring the TNF-α-content in the supernatants by ELISA after 6 h of co-culture. *p < 0.05 and **p < 0.01 *vs* the Ma-Mel-63a parental cell line (Student’s *t* test). (**C**) The parental Ma-Mel-63a cell line as well as the Ma-Mel-63a clones were subjected to protein extraction and subsequent western blotting using anti-p97/VCP and anti-β-actin (loading control) antibodies. The steady-state expression levels of p97/VCP in the parental Ma-Mel-63a cell line and the Ma-Mel-63a clones were visualized by densitometric analysis. The band intensity of p97/VCP was normalized to that of β-actin and subsequently expressed as fold-changes compared to the Ma-Mel-63a parental cell line which was set as 1.

The data obtained so far provide strong evidence that impaired expression of p97/VCP and subsequent dysfunction of the ERAD pathway was causative for the observed immune escape of CTL challenged UKVR-Mel-15a cells. Nevertheless, the possibility remained that this may represent an isolated case due to unknown specific features of the UKVR-Mel-15a cells. Therefore, we performed a second independent experimental analysis using the Melan-A/MART-1^+^, HLA-A*0201 melanoma cell line Ma-Mel-63a. As shown in Fig. 6A, Ma-Mel-63a cells expressed and efficiently presented the Melan-A/MART-1_26-35_ tumor epitope, as revealed by TNF-α-release assays. Importantly, overexpression of p97/VCP did not further improve the Melan-A/MART-1_26-35_ epitope presentation, suggesting that the ERAD pathway was fully functional in these cells. This notion was also supported by immunoblotting experiments revealing normal expression levels of the ERAD associated components (data not shown).

**Figure 7:**
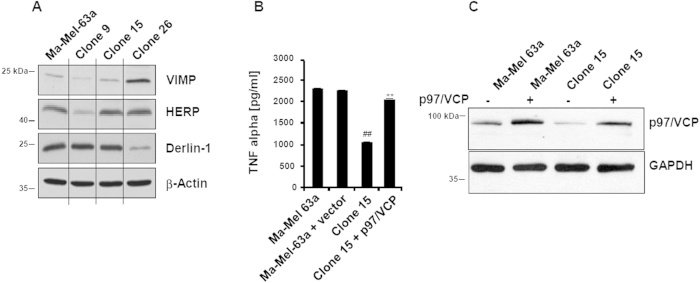
Melan-A/MART-1_26-35_ epitope presentation in the immune challenged Ma-Mel-63a clone 15 is rescued by p97/VCP over-expression. (**A**) The protein content of VIMP, HERP and Derlin-1 in the Ma-Mel-63a parental cell line and the Ma-Mel-63a clones 9, 15 and 26 was assessed by western-blotting, as indicated. Equal protein loading was ensured by probing the membrane with an anti-β-actin antibody. (**B**) The Ma-Mel-63a clone 15 was transfected with pcDNA3.1/p97/VCP for 24 h, as indicated and subsequently co-cultured with Melan-A/MART-1_26-35_ CTL for further 6 h. The ability of these cells to present the Melan-A/MART-1_26-35_ antigenic peptide was evaluated by measuring the content of TNF-α in the supernatants by ELISA. Control in this experiment consisted in the Ma-Mel-63a parental melanoma cell line transfected with the empty pcDNA3.1 plasmid or not. Shown is one representative experiment out of two. **p < 0.01 *vs* Ma-Mel-63a clone 15 and ^##^*p* < 0.01 *vs* Ma-Mel-63a cells (Student’s *t* test). (**C**) The Ma-Mel-63a clone 15 transfected or not with p97/VCP as well as the Ma-Mel-63a parental cell line were analyzed for their p97/VCP content by western-blotting, as indicated. GAPDH was used as loading control.

Following the same experimental conditions, Ma-Mel-63a cells were challenged twice in short-term co-culture with Melan-A/MART-1_26-35_-specific CTL. The Ma-Mel-63a clones resistant to CTL mediated lysis were further cultivated and assessed for their capacity of presenting the Melan-A/MART-1_26-35_ epitope. Of the 33 Ma-Mel-63a clones, 3 clones (i.e. clones 9, 15 and 26) exhibited substantial impaired recognition by Melan-A/MART-1_26-35_ CTL when compared with the unchallenged parental Ma-Mel-63a cell line (Fig. 6B). Strikingly, all three clones also displayed reduced p97/VCP expression (Fig. 6C). Of these, clone 26 could not be further analyzed because it lost its endogenous Melan-A/MART-1 expression (data not shown). The Ma-Mel-63a clone 15 was finally selected for p97/VCP rescue experiments, because it revealed an almost unaltered expression level of the ERAD components VIMP, HERP or Derlin-1 as well as a normal expression of the IFN-inducible i-subunits and TAP1/2 (Figs 7A and S4). As shown in Fig. 7B,C, overexpression of p97/VCP almost completely restored Melan-A/MART-1_26-35_ epitope presentation. These data show that impaired expression of the ERAD component p97/VCP upon immune challenge with Melan-A/MART-1_26-35_-specific T-cells is an universal event that allows melanoma cells to escape CTL-mediated cell death.

## Discussion

There is mounting clinical evidence that T-lymphocytes play a central role in the regression of melanoma *in vivo* and that adoptive transfer of cultured T cells from tumor infiltrating lymphocytes may result in tumor regression^31^. However, loss of MHC class I expression has been reported to render tumors resistant to adoptive T-cell therapy^32^. In fact, analysis of tumor cells also revealed a down-regulation of the IFN-γ-inducible antigen processing machinery^33^,^34^,^35^,^36^,^37^. It is not clear how frequent immune escape of tumors upon T cell pressure results in the irreversible loss of IFN-γ sensitivity. Studies of tumor cell lines showed that, in most cases, the expression of down-regulated components involved in MHC class I antigen presentation was restored by IFN-γ treatment. Nevertheless, immune evasion of tumor cells upon T cell pressure still remains a problem and very little is known about escape mechanism connected with antigen processing. Our experiments show that co-cultivation of the melanoma UKRV-Mel-15a and Ma-Mel-63a cells with Melan-A/MART-1_26-35_ CTL led in both instances to the isolation of several melanoma cell clones, which revealed impaired presentation of the Melan-A/MART-1_26-35_ antigenic peptide (Figs 1 and 6). However, immunoblot analyses failed to provide evidence that the expression of any of the known IFN-γ inducible components of the antigen processing and presentation machinery was significantly modified (Figs S1 and S4) and therefore did not explain the observed immune escape of the resistant UKRV-Mel-15a and Ma-Mel-63a clones.

The UPS with its more than 1000 protein components plays a central role in the generation of tumor epitopes presented by MHC class I molecules. Many of these may be directly or indirectly involved in the regulated proteasome dependent degradation of the Melan-A/MART-1 protein. Because of this complexity, we focused our analysis in particular on components of the ER associated degradation (ERAD) pathway known to be involved in the degradation of membrane-associated tumor antigens such as tyrosinase^38^. Indeed, our cDNA array analyses of melanoma cells resistant to cytolysis by Melan-A/MART-1_26-35_-specific CTL revealed several of the known ERAD components to be down regulated in these cells. Interestingly, the most reproducible down-regulation was observed for the AAA ATPase p97/VCP (Table 1). Importantly, the down-regulation of p97/VCP was observed in two independent melanoma cell lines, indicating that such effect was not cell line-specific but rather a more general phenomenon in response to CTL exposure (Figs 2A and 6C). The essential and critical role of ERAD in the ubiquitin-dependent degradation of membrane proteins and, as such, in the generation of antigenic peptide derived from membrane proteins is well documented^39^,^40^. Given the high number of membrane-bound proteins among melanoma-associated antigens, an active contribution of ERAD in tumor surveillance is very likely. For example, a functional ERAD pathway has been reported to be a prerequisite for the breakdown of the tyrosinase antigen^38^,^41^. This is also true for secretory proteins such as the pro-insulin auto-antigen^42^. Interestingly, the ERAD-mediated degradation of tyrosinase occurs in an EDEM1-dependent fashion, while that of pro-insulin mainly relies on Derlin-2, HRD1 and p97/VCP. The observation that ERAD substrates do not necessarily rely on the same components implies a strong heterogeneity and plasticity of the ERAD pathway. This point is of considerable importance, as it suggests that the loss of one particular ERAD-associated protein in tumors might not necessarily cause immune escape. An exception, however, is p97/VCP which is essentially required for the extraction of substrates from the ER to the cytoplasm, regardless of the composition of the ERAD complexes and/or pathways employed. Thus, any down-regulation of p97/VCP will be accompanied by a decreased MHC class I presentation of all ERAD-dependent antigens. Importantly, the involvement of ERAD in the supply of MHC class I-restricted peptides is not exclusively restricted to hydrophobic antigens. Hence, a major role for ERAD has also been described in the cross-presentation of soluble antigens by dendritic cells (DC)^43^,^44^,^45^,^46^, although the precise mechanisms by which internalized antigens use ERAD to gain access to the cytoplasm remain unclear. Interestingly, cross-presentation is not restricted to DC and may be also used by melanoma cells to present antigens derived from secretory proteins such as the matrix metalloproteinase-2 (MMP2)_560-568_ epitope^47^. As expected, a role for ERAD has been suggested in the processing of this antigenic peptide^48^, although the implication of p97/VCP remains to be formally addressed. Our experiments demonstrate that re-expression of p97/VCP in CTL-resistant UKRV-Mel-15a clone 18 and Ma-Mel-63a clone 15 to normal levels was able to almost completely recover Melan-A/MART-1_26-35_ epitope presentation. The expression of p97/VCP is not induced by cytokines and it is not clear at the moment why its expression may be so influenced by exposure of melanoma cells to Melan-A/MART-1_26-35_ CTL. In any case, the down-regulation of ERAD is of general importance, as it will affect not only the presentation of the Melan-A/MART-1_26-35_ epitope but also the presentation of all other membrane protein-derived tumor epitopes generated by the ERAD pathway. Our data suggest that, in order to minimize or circumvent rapid immune evasion upon T-cell therapy, those tumor epitopes should be chosen and simultaneously targeted by T cells that are derived from separate antigens, which are degraded and processed by distinct degradation pathways. In this context, the option to target antigens whose degradation does not primarily rely on the UPS appears indeed particularly attractive. In view of the UPS, and *a fortiori*, p97/VCP as a major target whose deregulation might facilitate tumor escape, future research efforts may also address the functional relevance of proteasome-independent MHC class I tumor peptides in immunotherapy.

## Material and Methods

### Cell culture and immune selection of melanoma cells

The T2 lymphoblastoid cell line as well as the human melanoma cell lines Ma-Mel-91 cells (Melan-A/MART-1^−^, HLA-A*0201), UKRV-Mel-15a (Melan-A/MART-1^+^, HLA-A*0201), Ma-Mel-63a (Melan-A/MART-1^+^, HLA-A*0201) and immune selected cell clones derived from UKRV-Mel-15a or Ma-Mel-63a were cultivated in RPMI supplemented with 10% fetal bovine serum (Biochrom AG, Germany). Authentication of these cell lines was ensured by genetic profiling on genomic DNA at the Institute for Forensic Medicine (University Hospital Essen) using the AmpFLSTR-Profiler Plus kit (Applied Biosystems)^49^. To obtain UKRV-Mel-15a or Ma-Mel-63a clones resistant to lysis by HLA-A*0201-restricted Melan-A/MART-1_26-35_-specific CTL, melanoma cells were co-cultured for 5 h with a Melan-A/MART-1_26-35_-specific CTL clone raised against the ELAGIGILTV mutant peptide at a ratio of 1:50. Resistant melanoma cells were kept in culture for two weeks, followed by one or two additional rounds of co-incubation with Melan-A/MART-1-specific CTL. UKRV-Mel-15a and Ma-Mel-63a cells were then cloned by limiting dilution at 0,3 cells/well in 96 well plates and single clones were expanded for further analysis.

### Preparation of RNA, microarray hybridization, and data analysis

Microarrays hybridizations were conducted, as previously described^50^. Briefly, total RNA was extracted, amplified and labelled with biotin (Message AmpII-Biotin enhanced Kit, Ambion). The Human U133 2.0 Plus-Array (Affymetrix) was custom hybridized and evaluated by standard procedures (Signature Diagnostics). Raw data files were deposited on NCBIs Gene Expression Omnibus (GEO, http://www.ncbi.nlm.nih.gov/geo/) under the accession number GSE75929.

### Transfection of melanoma cells for antigen presentation analysis

Melanoma cells were transfected with expression plasmids encoding pMART-1 wild-type, p97 wild-type, p97QQ, a dominant negative p97QQ mutant (both provided by T. Rapoport, Department of Cell Biology, Harvard Medical School, Boston, MA) (all cloned into pcDNA3.1 vector, Invitrogen)^16^ or control plasmid pcDNA3.1. Transfection was performed using Lipofectamine 2000 (Invitrogen) following manufacturer’s instructions. After 24–48 h of transfection, cells were harvested for further studies. For siRNA transfection, HighPerFect Reagent (Roche) was used. Cells were incubated for 72 h with ON-TARGET-plus SMART pools siRNA duplexes targeting HERP (human HERPUD1), p97/VCP or non-targeting (control) siRNA (all purchased from Dharmacon Inc.). VIMP-siRNA (5′-CTGGCGGATGAGGCTAAGAAT-3′) was purchased from Qiagen.

### Generation of Melan-A/MART-1_26-35_-specific CD8^+^T cell clone

A HLA-A*0201-restricted Melan-A/MART- 1-specific CD8^+^T cell clone was generated against the ELAGIGILTV mutant peptide as described previously^51^,^52^. For antigen presentation analysis, melanoma cells were incubated with Melan-A/MART-1-specific T cells for 6 h at an E:T ratio of 1:1 or 8:1, as previously described^53^. As positive control, target cells were loaded with 5 μM of the Melan-A/MART-1_26-35_ ELAGIGILTV peptide for 1 or 2 h. Activation of Melan-A/MART-1-specific CTL was assessed by quantification of secreted TNF-α or IFN-γ by ELISA as described below.

### ELISA

ELISA for TNF-α or IFN-γ was performed following the manufacturer’s instructions (BD Biosciences). Diluted (1:5) supernatants from CTL-experiments were added to 96 wells pre-coated with anti-TNF-α or anti-IFN-γ antibodies in duplicates and supplemented with secondary antibodies and streptavidin-bound POD for 1 h. Plates were washed and incubated for 30 min with TMB substrate (BD Biosciences). Optical density was determined at 450 nm.

### Immunoblot analysis

Cells were lysed in Mammalian Protein Extraction Reagent (Pierce) containing Complete Protease Inhibitors (Roche). Proteins were separated on SDS-PAGE and immunoblotted with antibodies specific for p97/VCP (provided by R. Hartmann-Petersen), VIMP, Derlin-1 (both provided by T. Rapoport), HERP (laboratory stock)^14^, GAPDH (Santa Cruz), Melan-A/MART-1 (Novocastra), β-actin (Santa Cruz), β5i/LMP7, β1i/LMP2, β2i/MECL-1 (laboratory stock), TAP1 (Rockland) and TAP2 (NBL). Detection was carried out by enhanced chemiluminescence. Densitometric analysis was performed using ImageJ NIG software.

### Flow cytometry analysis

HLA-A*0201 surface expression as well as intracellular Melan-A/MART-1 antigen expression was determined by flow cytometry using a FITC-conjugated mAb BB7.2 for HLA-*0201 and a Melan-A/MART-1 antibody (NovoCastra) followed by staining with FITC-conjugated anti-mouse antibody (BD Biosciences) for Melan-A/MART-1. Cells were analyzed on a Becton Dickinson FACSCalibur with Cell Quest software (BD Biosciences).

### CTL assay

After removing unbound peptides by three washing steps with PBS, Melan-A/MART-1 epitope presentation was determined in a TNF-α release assay. CTL-mediated lysis of resistant melanoma cell clone was investigated by incubating melanoma cells with 5 μM of the synthetic Melan-A/MART-1_26-35_ ELAGIGILTV peptide for 2 h, followed by a standard^51^ Cr release assay, as previously described^54^.

### Cycloheximide (CHX) chase

HeLa cells were exposed to either non-targeting (control) or p97/VCP siRNA for 24 h prior to a subsequent transfection with an expression plasmid encoding the Melan-A/MART-1 full-length protein. After 24 h of transfection, 50 μg/ml of CHX was added to all samples (0 h chase). Cells were collected after 1, 2, 4 and 8 h and Western blot analysis was performed for each time point using antibodies specific for p97/VCP, Melan-A/MART-1 and GAPDH (loading control).

## Additional Information

**How to cite this article**: Ebstein, F. *et al*. Exposure to Melan-A/MART-1_26-35_ tumor epitope specific CD8^+^ T cells reveals immune escape by affecting the ubiquitin-proteasome system (UPS). *Sci. Rep*. **6**, 25208; doi: 10.1038/srep25208 (2016).

## Supplementary Material

Supplementary Information

## Figures and Tables

**Table 1:** The immune challenge of the UKRV-Mel-15a melanoma cell line with Melan-A/MART-1_26-35_-specific CTL results in altered expression of ERAD-related genes.

Gene	Clone 6	Clone 18	Clone 30
p97/VCP	0.622	0.513	0.451
VIMP	0.768	0.656	0.779
HERP	0.852	0.742	1.675
Derlin-1	1.971	1.954	1.991

Following two rounds of exposure with Melan-A/MART-1_26-35_-specific CTL, the UKRV-Mel-15a melanoma cells resistant to cell lysis were further cultivated and cloned by limiting dilution. The transcription profile of the clones 6, 18 and 30 was analyzed by microarrays and compared to that of the untreated UKRV-Mel-15a parental cell line (shown is the expression ratio of the mRNA expression levels relative to that of the UKRV-Mel-15a parental cell line). Four genes belonging to the ERAD pathway (i.e. p97/VCP, VIMP, HERP and Derlin-1) were identified as being strongly altered in the UKRV-Mel-15a clones 6, 18 and 30.
