# Supplementary material for: Exposure to Melan-A/MART-126-35 tumor epitope specific CD8+T cells reveals immune escape by affecting the ubiquitin-proteasome system (UPS)
Source: Sci Rep. 2016 May 4;6:25208. doi: 10.1038/srep25208 (PMC4855237; doi:10.1038/srep25208)
Supplement: Supplementary Information [file srep25208-s1.pdf]

Supplemental information

Exposure to Melan-A/MART-1$_{26\text{-}35}$ tumor epitope specific CD8^{+} T-cells reveals immune escape by affecting the ubiquitin-proteasome system (UPS)

Frédéric Ebstein, Martin Keller, Annette Paschen, Peter Walden, Michael Seeger, Elke Bürger, Elke Krüger, Dirk Schadendorf, Peter- M. Kloetzel and Ulrike Seifert

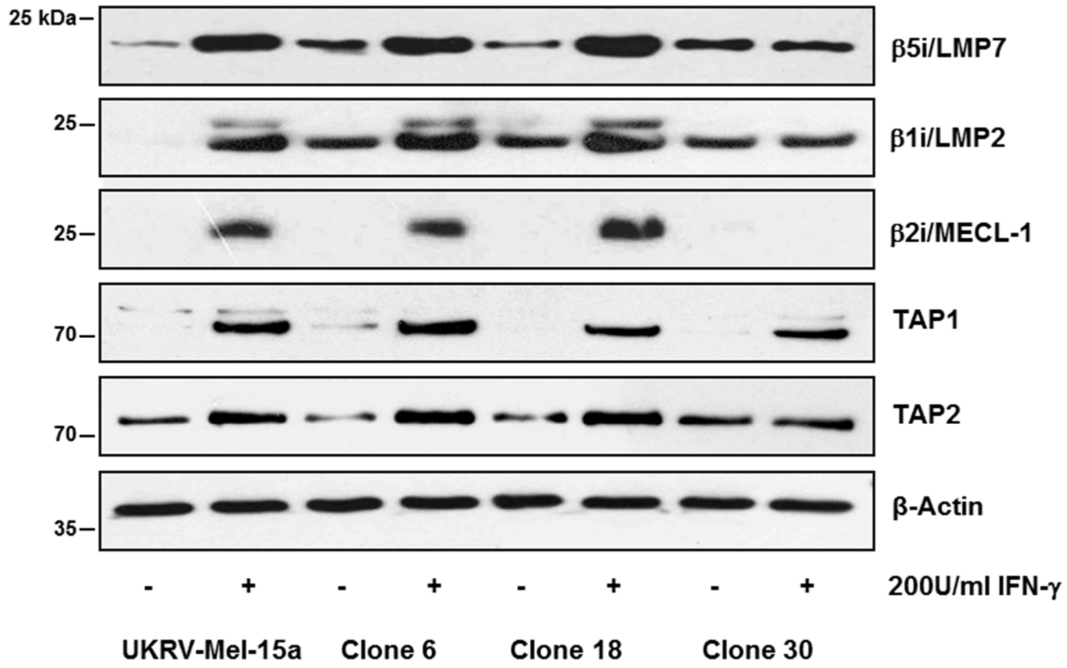

Figure S1. The immune challenge of the UKRV-Mel-15a melanoma cell line with Melan-A/MART-1$_{26\text{-}35}$ specific CTL is not accompanied by altered responsiveness to IFN-γ. Following two rounds of exposure with Melan-A/MART-1$_{26\text{-}35}$ specific CTL, the UKRV-Mel-15a melanoma cells resistant to cell lysis were further cultivated and cloned by limiting dilution. The UKRV-Mel-15a-derived clones 6, 18 and 30 as well as the UKRV-Mel-15a parental cell line were simulated with 200 U/ml IFN-γ for 48 h and subsequently collected and analysed by western-blotting for their content of β5i/LMP7, β1i/LMP2, β2i/MECL1, TAP1 and TAP2, as indicated. Equal protein loading was ensured by probing the membrane with an antibody specific for β-actin.

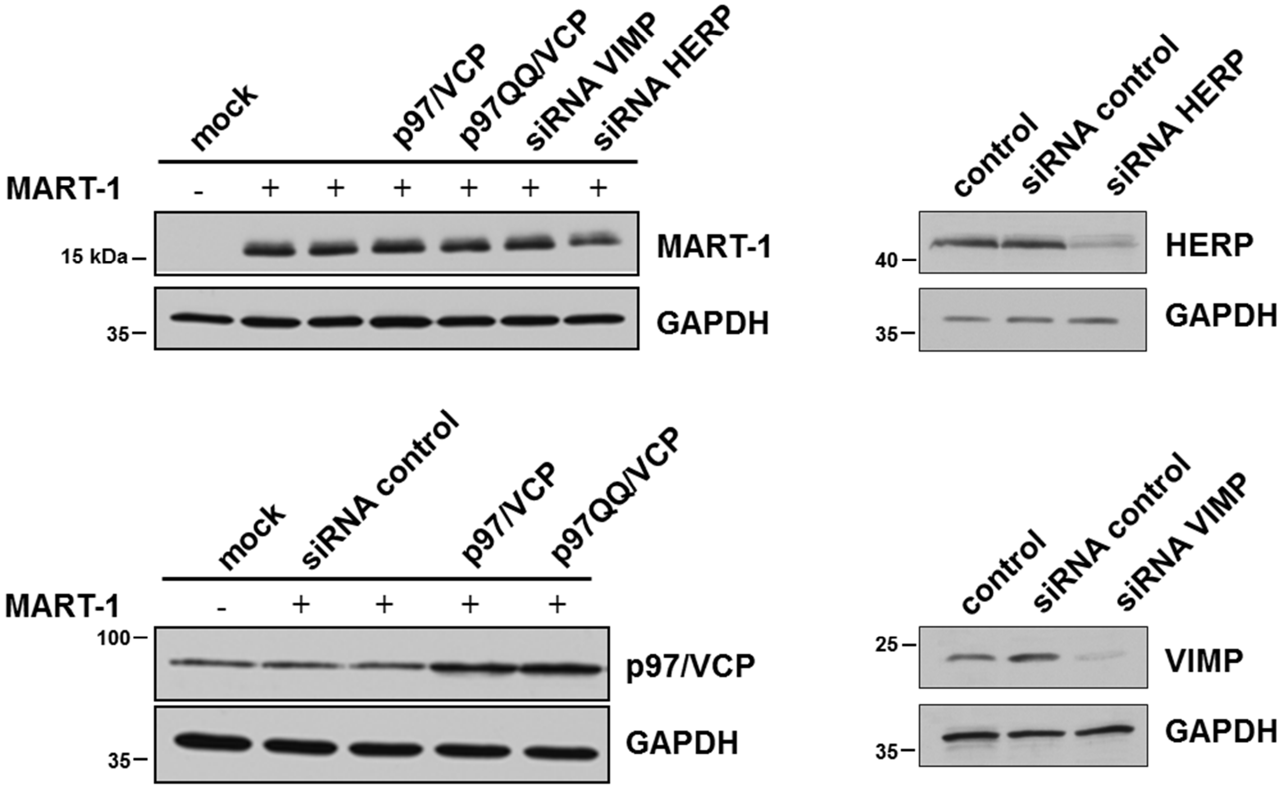

Figure S2. The remodelling of the p97/VCP, HERP and VIMP ERAD-related components in Mel91a cells does not alter the steady-state expression of the Melan-A/MART-1 full-length protein. Ma-Mel-91cells (Melan-A/MART-1$^-$, HLA-A*0201$^+$) were subjected to transfection with either a pcDNA3.1 empty vector or with Melan-A/MART-1 in combination with p97/VCP, p97QQ (p97/VCP neg), non-targeting (control) siRNA, HERP siRNA or VIMP siRNA. 24 h after overexpression or 72 h after siRNA treatment, cells were collected for protein extraction. Whole-cell extracts were resolved by SDS-PAGE followed by western blotting using antibodies specific for p97/VCP, VIMP, HERP and Melan-A/MART-1, as indicated. Equal protein loading was ensured by probing the membrane with anti-GAPDH antibody.

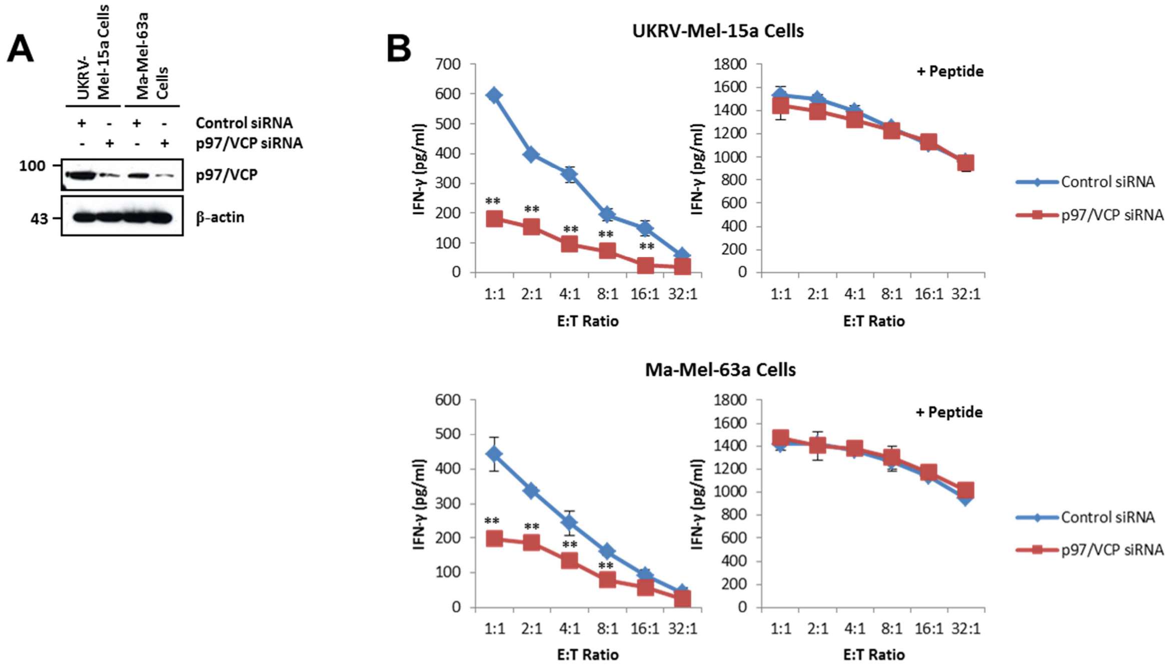

Figure S3. Melan-A/MART-1$_{26\text{-}35}$ epitope presentation in the UKRV-Mel-15a and Ma-Mel-63a parental cell lines critically relies on p97/VCP. (A) UKRV-Mel-15a and Ma-Mel-63a cells were exposed to a 72-h treatment with either non-targeting (control) or p97/VCP siRNA, as indicated. The protein content of p97/VCP in both of these cell lines was assessed by western-blotting using antibodies specific for p97/VCP and β-actin (loading control). (B) Endogenous presentation of the Melan-A/MART-1$_{26\text{-}35}$ antigenic peptide was evaluated by cultivating the UKRV-Mel-15a and Ma-Mel-63a cells with Melan-A/MART-1$_{26\text{-}35}$ specific CTL at various E:T ratio, as indicated. Control in this experiment consisted of UKRV-Mel-15a and Ma-Mel-63a exogenously loaded with 1 µM of the 10-mer Melan-A/MART-1$_{26\text{-}35}$ synthetic peptide. After 6 h, the supernatants were collected and tested for their IFN-γ content by ELISA. Shown is one representative experiment out of two. **$p<0.01$ (Student's *t* test).

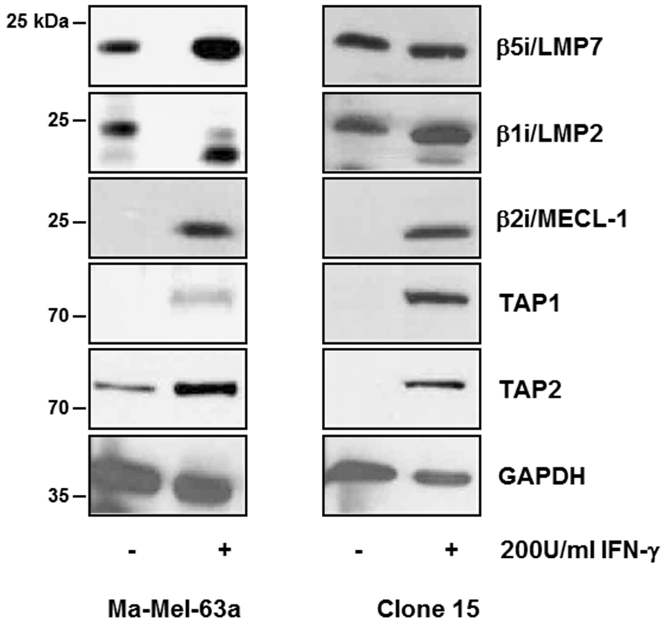

Figure S4. The immune challenge of the Ma-Mel-63a melanoma cell line with Melan-A/MART-1$_{26\text{-}35}$ specific CTL is not accompanied by altered responsiveness to IFN-γ. Following two rounds of exposure with Melan-A/MART-1$_{26\text{-}35}$ specific CTL, the Ma-Mel-63a melanoma cells resistant to cell lysis were further cultivated and cloned by limiting dilution. The Ma-Mel-63a-derived clone 15 and the Ma-Mel-63a parental cell line were simulated with 200 U/ml IFN-γ for 24 h and subsequently collected and analysed by western-blotting for their content of β5i/LMP7, β1i/LMP2, β2i/MECL1, TAP1 and TAP2, as indicated. Equal protein loading was ensured by probing the membrane with an antibody specific for GAPDH.
